# Prevalence and correlates of adherence to the combined movement guidelines among Czech children and adolescents

**DOI:** 10.1186/s12889-020-09802-2

**Published:** 2020-11-11

**Authors:** Lukáš Rubín, Aleš Gába, Jan Dygrýn, Lukáš Jakubec, Eliška Materová, Ondřej Vencálek

**Affiliations:** 1 Faculty of Physical Culture, Palacký University Olomouc, třída Míru 117, 771 11 Olomouc, Czech Republic; 2 Faculty of Science, Humanities and Education, Technical University of Liberec, Liberec, Czech Republic; 3 Faculty of Science, Palacký University Olomouc, Olomouc, Czech Republic

**Keywords:** 24-h movement guidelines, Associations, Family, Physical activity, Screen time, Sedentary behavior, Sleep, Youth

## Abstract

**Background:**

There are limited studies on the prevalence of adherence to the combined guidelines for physical activity (PA), sedentary behavior, and sleep in children and adolescents. Moreover, little is known about correlates of adherence to the guidelines. Therefore, the main aim of this study is to examine the prevalence and identify the correlates of adherence to the combined movement guidelines among children and adolescents.

**Methods:**

A total of 355 children aged 8–13 years (44% boys) and 324 adolescents aged 14–18 years (43% boys) from the Czech Republic participated in this study. PA and sleep duration were estimated using multi-day 24-h raw data from wrist-worn accelerometers. Recreational screen time was parent proxy-reported in children and self-reported in adolescents. Seventeen potential correlates were grouped into three homogenous categories for biological and cognitive, behavioral, and family correlates. The multi-level multivariable logistic regression was applied to identify correlates of adherence to combined movement guidelines and to specific combinations of any of two recommendations.

**Results:**

Approximately 6.5% of children and 2.2% of adolescents met all recommendations of the combined movement guidelines. In children, girls (OR = 0.4; 95% CI = 0.1–0.9) and participants with overweight or obese fathers (OR = 0.3; 95% CI = 0.1–0.7) had significantly lower odds of adherence to the combined movement guidelines. Additionally, children had higher odds of meeting specific combinations of two recommendations if they reported regular fruit and vegetable intake, participated in organized PA, or if their fathers had a university degree. Meanwhile, paternal overweight and obesity, and high sleep efficiency were associated with lower odds of meeting specific combinations of recommendations. In adolescents, sex, fruit and vegetable intake, organized PA, and active play were correlates of meeting specific combinations of any two recommendations.

**Conclusions:**

A low proportion of children and adolescents met the combined movement guidelines and several correlates related to family were identified. Family is a key source of influence for healthy movement behaviors during childhood and adolescence.

## Introduction

Physical activity (PA), sedentary behavior (SB), and sleep are key components of daily movement behaviors [1]. Ample evidence has confirmed the importance of all three behaviors (independently of each other) for physical, mental, and social health in children and adolescents [2–7]. Experts recommend that children and adolescents spend at least 60 min in moderate-to-vigorous PA (MVPA) [8], accumulate no more than 2 h of recreational screen time (ST) [9], and get enough sleep (9–11 h for children and 8–10 h for adolescents) every day [10] to maximize their health.

A favorable health status is associated with meeting at least two movement behavior recommendations rather than with meeting single recommendation [11–14]. To encourage healthy movement behaviors, the first guidelines for children and adolescents combining PA, SB, and sleep was released in 2016 in Canada [15]. Other countries, such as Australia [16], Croatia [17], and Thailand [18], later joined this effort. The World Health Organization (WHO) is now preparing global combined movement guidelines for children and adolescents in accordance with the WHO Global action plan on physical activity 2018–2030 [19].

Despite the confirmed health benefits associated with meeting the recommendations for PA, SB, and sleep, worldwide adherence to the combined movement guidelines is low among children and adolescents [20–26]. Unfortunately, over the last decades, scientific evidence has documented a negative secular trend for engaging in regular PA, limiting SB, and obtaining adequate sleep [27–29]. Consequently, it is reasonable to predict that the adherence to the combined movement guidelines will continue to decline in the near future unless effective interventions are developed and implemented.

Because of the low effectivity of interventions focusing on supporting a single movement behavior [30, 31], implementation of intervention strategies targeting all movement behaviors is essential for mitigating or reversing the current trends. Thus, identification of correlates of the combined movement guidelines is necessary to design effective multicomponent interventions. Although correlates of single movement recommendation have been identified [2, 32–36], there is, to our knowledge, a lack of studies focused on identifying correlates of the combined movement guidelines. A few studies have been published recently [21–24], but they included study samples with a narrow age range, used self-reported measurements of movement behaviors, and/or had a limited number of examined correlates. Therefore, the main objectives of this study are (1) to examine the prevalence of adherence to the combined movement guidelines, and (2) to identify the correlates of such guidelines among Czech children and adolescents.

## Methods

### Participants

Children (8–13 years) and adolescents (14–18 years) were recruited from 11 elementary and secondary schools. Schools with a specific focus on sport and schools for pupils with special educational needs were not included. Participants were recruited to participate on a voluntary basis via information flyers that were distributed through the school staff after the school management approved the research. The main inclusion criteria were participant age and good health condition. The participants whose parents reported medical complications that could affect PA and sleep were excluded from study. A total of 907 children and adolescents were enrolled in this study. Of all initial participants, 228 were excluded because they voluntarily withdrew from the study or became ill (*n* = 45), provided incomplete data (*n* = 129), their data could not be assessed due technical failures (*n* = 17), or did not meet accelerometer wear time criteria (*n* = 37). Hence, the final sample consisted of 355 children (44% boys) and 324 adolescents (43% boys). The detailed characteristics of the participants are shown in Table 1.

**Table 1:** Descriptive characteristics of children and adolescents

	Children ***n*** = 355				Adolescents ***n*** = 324				***p***-value^b^
	Mean	SD	Min	Max	Mean	SD	Min	Max	
**Personal data**									
Age (years)	11.7	1.6	8.1	13.9	16.3	1.3	14.0	18.0	< 0.001
Height (cm)	151.6	12.0	117.7	185.6	170.2	8.8	147.0	194.9	< 0.001
Weight (kg)	43.6	11.3	17.8	79.4	63.0	11.6	41.5	120.8	< 0.001
BMI *z*-score	0.24	1.13	−3.35	3.32	0.20	0.99	−2.83	3.49	0.587
**Movement behaviors**									
MVPA (min/day)^a^	58.1	24.3	7.2	151.7	39.3	19.1	2.9	100.7	< 0.001
ST (h/day)	3.0	1.8	0.1	11.9	2.8	2.1	0.1	12.9	0.206
Sleep duration (h/day)^a^	8.6	0.7	6.4	10.6	7.5	0.8	5.4	10.2	< 0.001

*BMI* Body mass index, *Max* Maximum, *Min* Minimum, *MVPA* Moderate-to-vigorous physical activity, *SD* Standard deviation, *ST* Screen time

^a^Accelerometer-based 24-h assessment; adjusted to 24 h before analysis

^b^The differences between age categories were analyzed using the *t*-test for independent samples

### Physical activity and sleep

PA and sleep were monitored using the wGT3X-BT and GT9X Link ActiGraph accelerometers (ActiGraph, Pensacola, FL, USA) worn by children and adolescents, respectively. The devices were initialized using the ActiLife software version 6.13.3 (ActiGraph, Pensacola, FL, USA), all three axes were used, and sampling interval was set to 100 Hz. To limit reactivity, the displays of GT9X Link accelerometers were set to show only date and time and the official start of monitoring was the next full day following the day on which the devices were distributed. Participants wore the activity monitor on their non-dominant wrist for 24 h over 7 consecutive days. They were instructed to remove the device only for swimming and bathing.

Raw accelerometer data were analyzed using the R-package GGIR version 1.10–7. Time spent in MVPA was estimated using the Hildebrand’s cut points for the Euclidian Norm Minus One metric [37]. Sleep duration (difference between sleep onset and waking up time) was calculated using the heuristic van Hees algorithm guided by participants’ sleep logs [38]. Sleep efficiency was calculated as the ratio of time spent in sustained inactivity periods divided by sleep duration. Only data from participants who had worn the accelerometer for at least 16 h per day for at least 4 days (including 1 weekend day) were included in the analyses. A more detailed description of PA and sleep assessment has been published elsewhere [39, 40].

### Screen time

Recreational ST was self-reported. A parent proxy report was required in children aged 12 years and younger (i.e., those in the first stage of elementary school). Participants, their parents, or guardians answered the questions taken from the questionnaire of the international Health Behaviour in School-aged Children study [41] as follows: *“About how many hours a day do you usually spend watching television, DVDs, videos (including YouTube or similar online service) in your free time on weekdays/weekend days?”* and *“About how many hours a day do you usually spend playing games on a computer, games console (PlayStation, Xbox, etc.), smartphone, tablet or similar electronic device in your free time on weekdays/weekend days?”*. Questions were separated for weekdays and weekend days. Nine different answers were available for each question (none, half an hour, 1, 2, 3, 4, 5, 6, and 7 or more hours a day). The validity and reliability of 7-day recall questions have been demonstrated in comparison with 7-day 24-h diaries both on weekdays and weekends [42]. Total amount of ST was calculated as the sum of weighted averages of ST during weekdays and weekend days.

### Adherence to the combined movement guidelines

Participants adhere to the combined movement guidelines if they accumulate at least 60 min of MVPA per day for PA recommendation, 2 h or less of recreational ST per day for SB recommendation, and 9–11 h per day for children and 8–10 h per day for adolescents for sleep recommendation.

### Correlates

Seventeen potential correlates were selected based on systematic reviews [2, 32–36] showing plausible associations with at least single recommendation included in the combined movement guidelines. Correlates were grouped into three categories: (1) biological and cognitive correlates, (2) behavioral correlates, and (3) family correlates. They were obtained through multiple research sources. Biological correlates except sex were measured directly using standard anthropometric measurements and the multi-frequency bioimpedance analyzer InBody 720 (InBody, Seoul, Korea). Cognitive and behavioral correlates were self-reported except for sleep efficiency, which was measured by accelerometry. Parent proxy report was required for participants aged 12 years and younger. Family correlates were reported by parents. The full list of correlates with information about their use in the analysis is displayed in Table 2.

**Table 2:** Potential correlates of meeting the combined movement guidelines

	*Method of measurement*	*Measurement/Question*	*Use in analysis*
**Biological and cognitive**			
Sex	Self-reported	Sex.	Binary variable: girl (1) or boy (0^a^)
School achievement	Self-reported	Did you pass with distinction on your final school report in the previous school year?	Binary variable: yes (1) or no (0^a^)
Adiposity	Device-measured	Assessed by multi-frequency bioimpedance analysis.	Re-coded as dichotomous: < 85th percentile (1^a^) or ≥ 85th percentile (0)
BMI *z*-score	Anthropometric measurement	World Health Organization BMI *z*-score based on direct measurement of body height and weight.	Re-coded as dichotomous: < 1 SD (1^a^) or ≥ 1 SD (0)
**Behavioral**			
Organized PA	Self-reported	About how many hours a week do you usually spend in organized sport activities in your free time on weekdays/weekend days?	Re-coded as dichotomous: ≥ 1 h a week (1) or < 1 h a week (0^a^)
Active play	Self-reported	About how many hours a day do you usually spend in unorganized PA in your free time on weekdays/weekend days?	Re-coded as dichotomous: ≥ 2 h a day (1) or < 2 h a day (0^a^)
AT to school	Self-reported	Check the prevailing mode (walk, bicycle, in-line, skateboard, car, bus, train) of transportation on the way to school.	Re-coded as dichotomous: ≥ 3 days a week of AT (1) or < 3 days a week of AT (0^a^)
AT from school	Self-reported	Check the prevailing mode (walk, bicycle, in-line, skateboard, car, bus, train) of transportation on the way from school.	Re-coded as dichotomous: ≥ 3 days a week of AT (1) or < 3 days a week of AT (0^a^)
Sleep efficiency	Device-measured	Measured by accelerometry and defined as the ratio of time spent in sustained inactivity periods divided by sleep duration.	Re-coded as dichotomous: ≥ 0.85 (1) or < 0.85 (0^a^)
Fruit and vegetable intake	Self-reported	About how many times a week do you usually eat or drink a) fruits and b) vegetables?	Re-coded as dichotomous: ≥ 1 day a week (1) or < 1 day a week (0^a^) for “a” and “b”
Unhealthy snacking	Self-reported	About how many times a week do you usually eat or drink c) sweets (candy or chocolate), d) coke or other soft drinks that contain sugar, and e) crisps, chips, salt sticks, etc.?	Re-coded as dichotomous: ≥ 1 day a week (1) or < 1 day a week (0^a^) for “c”, “d” or “e”
Skipping breakfast	Self-reported	How often do you usually have breakfast (more than a glass of milk or fruit juice) on weekdays/weekend days?	Re-coded as dichotomous: < 4 days a week (1) or ≥ 4 days a week (0^a^)
**Family**			
Maternal BMI	Parent-reported	Calculated from the self-reported body height and weight.	BMI computed and re-coded as dichotomous: < 25 kg/m^2^ (1^a^) or ≥ 25 kg/m^2^ (0)
Maternal education	Parent-reported	Highest educational level.	Re-coded as dichotomous: university and higher education (1) or lower than university education (0^a^)
Paternal BMI	Parent-reported	Calculated from the self-reported body height and weight.	BMI computed re-coded as dichotomous: < 25 kg/m^2^ (1^a^) or ≥ 25 kg/m^2^ (0)
Paternal education	Parent-reported	Highest educational level.	Re-coded as dichotomous: university and higher education (1) or lower than university education (0^a^)
Family income	Parent-reported	Is the total gross income of your household greater than 48,000 CZK per month (twice the median in Czechia)?	Binary variable: yes (1) or no (0^a^)

Parent proxy report was required for participants aged 12 years and younger

*AT* Active travel, *BMI* Body Mass Index, *PA* Physical activity, *SD* Standard deviation

^a^indicates reference category

### Procedure

Data were collected from 2018 to 2019 during regular school weeks. Participants were given accelerometers in the classrooms and were instructed on how to wear them properly and how to complete relevant sleep logs. Participants and/or their parents or guardians were asked to fill in the questionnaires. Parents or guardians responded to family characteristic questions. Participants responded to the remaining questions (except children aged 12 years and younger where answering by parents was required). After 8 days, accelerometers, sleep logs, and questionnaires were collected.

### Statistical analyses

Statistical analyses were conducted using the IBM SPSS Statistics version 23 (IBM, Armonk, NY, USA) and R version 3.4.2 (R Foundation for Statistical Computing, Vienna, Austria). All analyses were performed separately for children and adolescents. The differences between children and adolescents were analyzed using the *t*-test for continuous variables and the chi-squared test for categorical variables.

Univariable analysis was conducted to examine associations between potential correlates and adherence to the combined movement guidelines and the specific combinations of any two recommendations. Binary logistic regression models were used because of the inherent nature of dependent variables (“0” for not meeting and “1” for meeting the combined movement guidelines or combinations of any two recommendations). If an explanatory variable reached a less-strict criterion level of *p* < 0.1, it was retained for further analysis to prevent the exclusion of potentially important correlates.

Multi-level multivariable logistic regression analysis was performed to identify correlates of adhering to the combined movement guidelines and of meeting combinations of any two recommendations. The potential correlates were included in the final models as fixed effects (Level 1), while the school location was considered a random effect (Level 2) in all mixed effects models. The necessity to include the factor of school location in the model was tested (by the likelihood-ratio test) and the factor was omitted whenever possible. Odds ratios (OR) and the 95% confidence intervals (CI) corresponding to the individual correlates as well as their significance were calculated. The forward selection method was used to set up the final model. The final models include all correlates whose omission would lead to a significant decrease in the Akaike information criterion. All statistical analyses were conducted at a significance level of *p* < 0.05.

## Results

Descriptive characteristics of the participants are shown in Table 1. Significant differences between children and adolescents were found for all movement behaviors except ST. Children engaged in more MVPA (18.8 min/day) and slept longer (65.3 min/day) than adolescents (*p* < 0.001 for both).

**Fig. 1:**
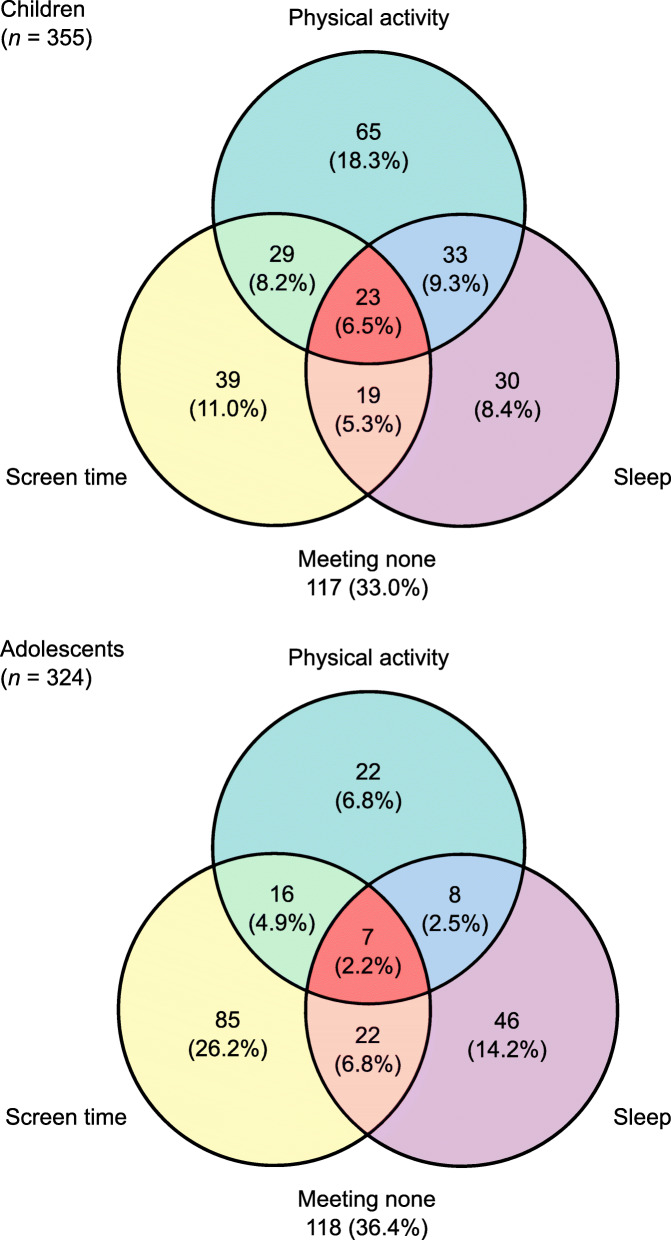
Prevalence of adherence to the combined movement guidelines in children and adolescents

Figure 1 displays the prevalence of adherence to the combined movement guidelines or its specific combinations among children and adolescents. Children met the combined movement guidelines (6.5%) in higher proportion (*p* = 0.006) compared with adolescents (2.2%). More specifically, children have significantly higher adherence to the PA alone recommendation (18.3 vs 6.8%; *p* < 0.001) and the combination of PA and sleep recommendations (9.3 vs 2.5%; *p* < 0.001) than adolescents. Children met the ST alone (11.0 vs 26.2%; *p* < 0.001) and sleep alone (8.4 vs 14.2%; *p* = 0.018) recommendations in lower proportion than adolescents.

Tables 3 and 4 present the univariable analysis of associations between selected correlates and adhering to the combined movement guidelines or meeting any two recommendations. In the univariable analysis, twelve and six potential correlates reached a *p*-value of less than 0.1 for at least one combination of meeting movement recommendations in children and adolescents, respectively. These potential correlates were retained for further analysis.

**Table 3:** Univariable analysis of correlates of meeting the combined movement guidelines in children (*n* = 355)

	*n (%)*^*a*^	PA + ST + SL			PA + ST			PA + SL			ST + SL		
		OR	95% CI	*p*-value	OR	95% CI	*p*-value	OR	95% CI	*p*-value	OR	95% CI	*p*-value
**Biological and cognitive**													
Sex	355 (44)	0.4	0.2–0.9	0.037	0.8	0.5–1.5	0.488	0.7	0.4–1.2	0.183	0.9	0.5–1.8	0.826
School achievement	349 (24)	1.5	0.5–5.2	0.507	2.1	1.0–5.4	0.078	2.1	1.0–4.9	0.077	2.5	1.0–7.4	0.066
Adiposity	355 (86)	0.3	0.0–1.3	0.204	0.3	0.1–1.0	0.082	0.6	0.2–1.4	0.255	0.3	0.1–1.0	0.089
BMI *z*-score	355 (75)	0.3	0.0–1.0	0.087	0.5	0.2–1.1	0.103	0.6	0.3–1.3	0.211	0.5	0.2–1.1	0.108
**Behavioral**													
Organized PA	351 (28)	1.4	0.6–4.4	0.496	1.2	0.6–2.4	0.611	2.3	1.1–5.1	0.035	1.8	0.8–4.2	0.176
Active play	355 (25)	1.2	0.5–3.7	0.750	1.7	0.8–3.8	0.195	1.6	0.8–3.5	0.211	1.2	0.6–2.8	0.622
AT to school	342 (52)	0.8	0.3–1.8	0.513	1.1	0.6–1.9	0.833	1.1	0.6–1.9	0.817	0.9	0.4–1.7	0.702
AT from school	338 (41)	0.6	0.2–1.3	0.181	0.9	0.5–1.7	0.716	0.9	0.5–1.6	0.643	0.6	0.3–1.2	0.119
Sleep efficiency	343 (40)	0.5	0.2–1.3	0.154	0.7	0.4–1.3	0.262	0.3	0.2–0.6	< 0.001	0.6	0.3–1.2	0.119
Fruit and vegetable intake	355 (67)	2.3	1.0–5.5	0.052	2.1	1.2–3.8	0.015	1.8	1.0–3.2	0.050	2.5	1.3–4.8	0.006
Unhealthy snacking	355 (58)	1.3	0.5–3.0	0.557	0.7	0.4–1.3	0.246	2.3	1.3–4.1	0.006	1.3	0.7–2.3	0.431
Skipping breakfast	350 (81)	0.2	0.0–1.0	0.115	0.4	0.1–1.0	0.089	0.7	0.3–1.6	0.442	0.3	0.1–0.9	0.068
**Family**													
Maternal BMI	338 (66)	0.5	0.2–1.4	0.242	0.7	0.3–1.3	0.256	0.8	0.4–1.4	0.429	0.7	0.3–1.4	0.284
Maternal education	346 (58)	1.2	0.5–2.8	0.706	2.0	1.1–3.7	0.028	0.6	0.3–1.1	0.081	1.2	0.6–2.4	0.514
Paternal BMI	328 (27)	0.3	0.1–0.7	0.005	0.4	0.2–0.8	0.010	0.6	0.4–1.2	0.165	0.3	0.2–0.7	0.002
Paternal education	334 (58)	2.1	0.9–5.2	0.097	3.1	1.7–5.9	0.001	1.0	0.6–1.9	0.912	1.9	1.0–3.9	0.054
Family income	284 (36)	2.0	0.7–7.4	0.219	1.6	0.8–3.4	0.196	1.4	0.7–3.0	0.339	0.9	0.4–1.8	0.691

*AT* Active travel, *BMI* Body mass index, *CI* Confidence interval, *OR* Odds ratio, *PA* Physical activity, *SL* Sleep, *ST* Screen time

^a^ Number of children included in the regression model and their proportion in the reference category

**Table 4:** Univariable analysis of correlates of meeting the combined movement guidelines in adolescents (*n* = 324)

	*n (%)*^*a*^	PA + ST + SL			PA + ST			PA + SL			ST + SL		
		OR	95% CI	*p*-value	OR	95% CI	*p*-value	OR	95% CI	*p*-value	OR	95% CI	*p*-value
**Biological and cognitive**													
Sex	324 (43)	4.6	0.6–38.9	0.158	2.9	1.0–8.0	0.041	0.6	0.2–1.8	0.406	2.5	1.1–6.1	0.038
School achievement	320 (66)	5.0	1.1–35.0	0.058	2.0	0.8–4.9	0.116	1.7	0.6–4.9	0.310	1.9	0.9–4.1	0.103
Adiposity	324 (86)	1.1	0.1–6.4	0.956	1.0	0.2–2.9	0.938	1.6	0.4–5.4	0.461	0.7	0.2–2.2	0.596
BMI *z*-score	324 (80)	1.6	0.3–7.7	0.573	0.8	0.2–2.3	0.740	1.5	0.4–4.5	0.515	0.4	0.1–1.3	0.182
**Behavioral**													
Organized PA	323 (43)	NA	NA	NA	2.2	0.9–6.3	0.102	3.1	1.0–13.9	0.083	1.7	0.8–4.1	0.187
Active play	324 (43)	1.9	0.4–13.6	0.437	1.0	0.4–2.4	0.978	5.3	1.4–33.9	0.031	1.5	0.7–3.5	0.323
AT to school	313 (66)	0.8	0.1–3.7	0.765	1.1	0.4–2.7	0.797	0.9	0.2–2.7	0.810	1.2	0.5–2.6	0.628
AT from school	308 (53)	0.5	0.1–2.1	0.342	1.0	0.4–2.3	0.899	0.5	0.1–1.6	0.247	0.9	0.4–2.0	0.827
Sleep efficiency	319 (26)	0.9	0.2–6.3	0.892	0.8	0.3–2.2	0.643	0.5	0.2–1.6	0.225	0.7	0.3–1.8	0.466
Fruit and vegetable intake	324 (67)	5.3	1.1–37.2	0.049	2.9	1.2–6.9	0.016	2.4	0.9–7.1	0.096	1.5	0.7–3.2	0.318
Unhealthy snacking	324 (75)	NA	NA	NA	0.8	0.3–2.1	0.683	1.5	0.5–4.4	0.467	1.1	0.5–2.6	0.768
Skipping breakfast	322 (77)	0.5	0.0–3.2	0.574	0.5	0.1–1.4	0.237	0.5	0.1–1.8	0.359	1.1	0.4–2.5	0.910
**Family**													
Maternal BMI	306 (61)	NA	NA	NA	0.7	0.3–1.6	0.407	0.4	0.1–1.4	0.190	0.5	0.2–1.2	0.128
Maternal education	316 (60)	1.1	0.2–5.2	0.871	1.0	0.4–2.3	0.940	0.7	0.2–2.2	0.597	1.7	0.8–3.7	0.175
Paternal BMI	286 (26)	2.1	0.4–39.8	0.499	0.5	0.2–1.2	0.091	1.2	0.3–5.2	0.836	0.9	0.4–2.2	0.788
Paternal education	295 (58)	1.8	0.4–9.5	0.430	1.9	0.8–4.8	0.161	1.4	0.5–4.1	0.556	1.3	0.6–2.8	0.499
Family income	235 (35)	2.2	0.3–42.9	0.490	0.8	0.3–2.4	0.669	0.5	0.2–1.7	0.267	1.4	0.5–4.0	0.526

NA indicate insufficient sample size for estimation

*AT* Active travel, *BMI* Body mass index, *CI* Confidence interval, *OR* Odds ratio, *PA* Physical activity, *SL* Sleep, *ST* Screen time

^a^ Number of adolescents included in the regression model and their proportion in the reference category

The results of the multi-level multivariable analysis are shown in Table 5. In children, girls have significantly lower odds of adherence to the combined movement guidelines than boys (OR = 0.4; 95% CI = 0.1–0.9). Children have significantly lower odds of adherence to the combined movement guidelines if their father is overweight or obese (OR = 0.3; 95% CI = 0.1–0.7). Moreover, fruit and vegetable intake was associated with meeting the combinations of PA and ST (OR = 2.0, 95% CI = 1.1–3.8), and ST and sleep recommendations (OR = 2.7, 95% CI = 1.3–5.5). Children that participated in organized PA (OR = 2.5, 95% CI = 1.1–5.7) had higher odds of meeting the combination of PA and sleep recommendations, while children with high sleep efficiency (OR = 0.4, 95% CI = 0.2–0.7) had lower odds of meeting the same combination of recommendations. Paternal overweight and obesity was associated with lower odds of meeting the combinations of PA and ST (OR = 0.5, 95% CI = 0.4–1.0), and ST and sleep recommendations (OR = 0.4, 95% CI = 0.2–0.8). Children had significantly higher odds of meeting the combination of PA and ST recommendations (OR = 2.8, 95% CI = 1.5–5.4) if their fathers had a university degree. In adolescents, those who reported regular fruit and vegetable intake had higher odds of meeting combination of PA and ST recommendations (OR = 2.9, 95% CI = 1.2–7.3). Adolescents who participated in organized PA and active play had higher odds of meeting the combinations of PA and ST (OR = 2.9, 95% CI = 1.1–9.3), and PA and sleep recommendations (OR = 5.1, 95% CI = 1.4–32.7), respectively. Adolescent girls have significantly higher odds of meeting the combinations of ST and sleep recommendations (OR = 2.5, 95% CI = 1.1–6.5) than adolescent boys.

**Table 5:** Multi-level multivariable analysis of correlates of meeting the combined movement guidelines in children and adolescents

	Children ***n*** = 355					Adolescents ***n*** = 324			
	*n (%)*^*a*^	OR	95% CI	*p*-value		*n (%)*^*a*^	OR	95% CI	*p*-value
**PA + ST + SL**					**PA + ST + SL**				
Sex	328 (45)	0.4	0.1–0.9	0.037	School achievement	320 (66)	4.8	1.0–33.9	0.066
Fruit and vegetable intake	328 (65)	2.2	0.9–5.6	0.079	Fruit and vegetable intake	320 (67)	5.1	1.1–36.0	0.057
Paternal BMI	328 (27)	0.3	0.1–0.7	0.006					
**PA + ST**					**PA + ST**				
Fruit and vegetable intake	325 (65)	2.0	1.1–3.8	0.034	Fruit and vegetable intake	285 (68)	2.9	1.2–7.3	0.019
Paternal BMI	325 (28)	0.5	0.4–1.0	0.034	Organized PA	285 (43)	2.9	1.1–9.3	0.043
Paternal education	325 (58)	2.8	1.5–5.4	0.002	Paternal BMI	285 (26)	0.4	0.2–1.1	0.066
**PA + SL**^b^					**PA + SL**				
School achievement	335 (23)	2.1	0.8–5.4	0.131	Organized PA	323 (43)	3.0	0.9–13.4	0.096
Organized PA	335 (28)	2.5	1.1–5.7	0.038	Active play	323 (43)	5.1	1.4–32.7	0.036
Sleep efficiency	335 (40)	0.4	0.2–0.7	0.003					
Unhealthy snacking	335 (58)	1.9	1.0–3.8	0.051					
**ST + SL**					**ST + SL**				
School achievement	322 (23)	3.3	1.1–14.0	0.060	Sex	320 (43)	2.5	1.1–6.5	0.041
Fruit and vegetable intake	322 (65)	2.7	1.3–5.5	0.006	School achievement	320 (66)	1.9	0.9–4.1	0.114
Paternal BMI	322 (28)	0.4	0.2–0.8	0.009					

*AT* Active travel, *BMI* Body mass index, *CI* Confidence interval, *OR* Odds ratio, *PA* Physical activity, *SL* Sleep, *ST* Screen time

^a^ Number of participants included in the model and their proportion in the reference category

^b^ Response variable was associated with school location; multi-level regression model was used

## Discussion

The present study revealed that a low proportion of Czech children and adolescents met the combined movement guidelines for PA, SB and sleep. Children met the combined movement guidelines in higher proportion compared with adolescents. We found that sex and paternal overweight and obesity were associated with adherence to the combined movement guidelines in children. Several correlates of combinations of any two recommendations have been identified for both age categories.

To the best of our knowledge, the accelerometry-based estimates of MVPA and sleep for evaluating adherence to movement guidelines were used in two studies that included children aged 9–11-years from 13 countries [20, 21]. Compared with these countries, the proportion of Czech children who met the combined movement guidelines is below average, and this result is comparable to the prevalence in middle- (India and Kenya) and high-income (Finland) countries. However, the differences in adherence to movement guidelines between our study and the aforementioned studies should be interpreted with caution due to discrepancies in the ages of the participants and approaches used to obtain and analyze accelerometer data.

The novel finding of the present study is that adherence to the combined movement guidelines was sex-specific and paternal overweight and obesity lowered odds of meeting all movement behavior recommendations in children. To this date, only four studies were focused on correlates of the combined movement guidelines [21–24]. The parental weight status was analyzed only in the study by Manyanga and colleagues [21], who found no association with the adherence to the combined movement guidelines. However, previous studies have shown that parental weight status is associated with children’s movement behaviors. Angoorani and colleagues [43] found higher odds of having low PA level and high ST in children whose parents were overweight or obese. One possible explanation for our results could be that parental obesogenic behaviors and shared home environment might be associated with movement behaviors. For example, an insufficient PA level of fathers is associated with a low PA level of their children [44] and/or presence of screens in bedroom is associated with greater ST and lower sleep duration [45]. For this reason, interventions to promote healthy movement behaviors during childhood need to involve both parents and should take into account the shared home environment.

The present study identified several correlates associated with meeting the specific combinations of any two recommendations. The participation in organized PA and active play was associated with meeting the combinations of PA and ST, and PA and sleep recommendations. This finding illustrates the compensatory change between the 24-h movement behaviors, which are typical examples of compositional data [1]. We can hypothesize that participation in organized and unorganized PA leads to an increase in overall PA, which results in compensatory changes in the remaining movement behaviors. Previous studies support this assumption by showing that a greater amount of time spent engaged in PA is associated with lower ST and longer sleep duration [46, 47]. Furthermore, we found that fruit and vegetable intake, parental characteristics, and sleep efficiency were also associated with specific combinations of recommendations. Similar to the participation in organized PA and active play, these correlates are related to the family, which represents the key source of influence in lifestyle behaviors of children and adolescents [48]. The importance of family for meeting movement behavior recommendations in children and adolescents is supported by the recent study by Chen and colleagues [24], who found that parental educational level and family income are significantly positively associated with adherence to the combined movement guidelines.

Our study showed that the prevalence and correlates of adherence to the combined movement guidelines or meeting specific combinations of any two recommendations differ between children and adolescents. Similar to our study, Roberts et al. [49] found that children have significantly higher prevalence of meeting the combined movement guidelines compared with adolescents. These findings illustrate the age-related changes in movement behaviors that have been previously documented [50, 51]. We may have identified different types and number of correlates between children and adolescents because the family influence changes during the transition from childhood to adolescence. For example, adolescents have higher bed time autonomy than children [52], which could result in longer late-night ST and short sleep duration. Alternatively, adolescents may spend more of their free time outdoors without parental supervision, which could explain the association between adolescents’ active play and meeting the specific combination of the combined movement guidelines.

The main strength of the present study is the multi-day 24-h accelerometer-based assessment and raw data processing to estimate the amount of time spent in MVPA and sleep duration. Additionally, wrist-worn accelerometers provide more valid and comparable data as a result of increased participant compliance, reduction of non-wear time [53], and more precise estimates of sleep duration compared with hip-worn devices [54]. Another strength is the relatively large sample size that included participants with a wide age range. The use of multi-level multivariable regression could also be considered one of the strengths of this study.

This study has some limitations that must be mentioned. First, we were unable to determine the causality of the associations due to the cross-sectional design. Second, the low percentages of adherence to the combined movement guidelines and to any two of its components require cautious interpretation of *p*-values because all the tests performed are asymptotic and only approximate. Third, the associations found are limited to the list of potential correlates. It is necessary to mention that environmental correlates (except family environment) have not been examined in the present study. Future studies should examine more potential environmental correlates because, according to socio-ecological models, they are associated with movement behaviors. Fourth, the potential correlates included in this study were mostly self- or parent-reported. Fifth, recreational ST was parent proxy-reported in children and self-reported in adolescents which might produce different estimates of ST between age groups. Finally, the results are not fully generalizable to other young populations because the correlates may not be similar across different cultures [55].

## Conclusions

The present study revealed that a low proportion of Czech children and adolescents met the combined movement guidelines. Children have a higher prevalence of meeting all three recommendations included in the combined movement guidelines than adolescents. Sex of participants and paternal weight status were the only correlates associated with meeting the combined guidelines in children. Several correlates for specific combinations of any two recommendations have been found in both age categories. Family is related to all identified correlates and plays a crucial role in healthy lifestyle during childhood and adolescence. To design effective interventions supporting adherence to the combined movement guidelines among children and adolescents, family environment including parental characteristics should be considered.

## Data Availability

The dataset analyzed during the current study is available in the Figshare repository, 10.6084/m9.figshare.12680855.

## Abbreviations

CI: Confidence interval
MVPA: Moderate-to-vigorous physical activity
OR: Odds ratio
PA: Physical activity
SB: Sedentary behavior
SD: Standard deviation
ST: Screen time
WHO: World Health Organization
